# Serum Selenium and Incident Cardiovascular Disease in the PREvención con DIeta MEDiterránea (PREDIMED) Trial: Nested Case-Control Study

**DOI:** 10.3390/jcm11226664

**Published:** 2022-11-10

**Authors:** Mario Gutiérrez-Bedmar, Fernando Gil, Pablo Olmedo, Miguel Ruiz-Canela, Miguel Ángel Martínez-González, Jordi Salas-Salvadó, Nancy Babio, Montserrat Fitó, Jose Luís Del Val García, Dolores Corella, José V. Sorlí, Emilio Ros, Miquel Fiol, Ramón Estruch, José Manuel Santos-Lozano, Fernando Arós, Lluís Serra-Majem, Xavier Pintó, Enrique Gómez-Gracia, Carlos Muñoz-Bravo

**Affiliations:** 1 Preventive Medicine and Public Health Department, School of Medicine, University of Málaga, 29010 Malaga, Spain; 2 Biomedical Research Institute of Malaga-IBIMA Plataforma BIONAND, 29010 Malaga, Spain; 3 CIBERCV Cardiovascular Diseases, Carlos III Health Institute, 28029 Madrid, Spain; 4 Department of Legal Medicine, Toxicology, and Physical Anthropology, School of Medicine, University of Granada, 18071 Granada, Spain; 5 Department of Preventive Medicine and Public Health, School of Medicine, University of Navarra, 31008 Pamplona, Spain; 6 CIBEROBN Physiopathology of Obesity and Nutrition, Carlos III Health Institute, 28029 Madrid, Spain; 7 Department of Nutrition, Harvard T.H. Chan School of Public Health, Boston, MA 02115, USA; 8 Human Nutrition Unit, Departament de Bioquímica i Biotecnologia, Universitat Rovira I Virgili, Hospital Universitari San Joan de Reus, 43201 Reus, Spain; 9 Institut d’Investigació Sanitària Pere Virgili (IISPV), 43201 Reus, Spain; 10 Unit of Cardiovascular Risk and Nutrition, Hospital del Mar Institute for Medical Research (IMIM), 08003 Barcelona, Spain; 11 Unitat d’Avaluació, Sistemes d’Informació i Qualitat (BASIQ), Gerència Territorial Atenció Primària Barcelona Ciutat, Institut Català de la Salut, 08029 Barcelona, Spain; 12 Fundació Institut Universitari per a la Recerca a l’Atenció Primària de Salut Jordi Gol I Gurina (IDIAPJGol), 08007 Barcelona, Spain; 13 Department of Preventive Medicine, Universidad de Valencia, 46010 Valencia, Spain; 14 August Pi i Sunyer Biomedical Research Institute (IDIBAPS), 08036 Barcelona, Spain; 15 Lipid Clinic, Endocrinology and Nutrition Service, Hospital Clínic, 08036 Barcelona, Spain; 16 Health Research Institute of the Balearic Islands (IdISBa), Hospital Son Espases, 07120 Palma de Mallorca, Spain; 17 Internal Medicine Service, Hospital Clínic, 08036 Barcelona, Spain; 18 Research Unit, Department of Family Medicine, Distrito Sanitario Atención Primaria Sevilla, 41010 Sevilla, Spain; 19 Department of Cardiology, Hospital Txagorritxu, 01009 Vitoria, Spain; 20 Department Clinical Sciences, University of Las Palmas de Gran Canaria, 35016 Palmas de Gran Canaria, Spain; 21 Lipid Unit, Department of Internal Medicine, IDIBELL-Hospital Universitari de Bellvitge, L’Hospitalet de Llobregat, FIPEC, 08908 Barcelona, Spain

**Keywords:** cardiovascular disease, serum selenium, older populations, PREDIMED, mediterranean diet

## Abstract

Background: Selenium is an essential trace mineral with potential interest for cardiovascular disease (CVD) prevention owing to its antioxidant properties. Epidemiological data on selenium status and CVD remain inconsistent. The objective of this study was to ascertain whether low serum selenium (SSe) concentrations are related to an increased risk of a first CVD event in a population at high cardiovascular risk. Methods: We undertook a case-control study nested within the “PREvención con DIeta MEDiterránea” (PREDIMED) trial. A total of 207 participants diagnosed with CVD (myocardial infarction, stroke, or cardiovascular death) during the follow-up period (2003–2010) were matched by sex, age, and intervention group to 436 controls by incidence density sampling. Median time between serum sample collection and subsequent CVD event occurrence was 0.94 years. SSe levels were determined using inductively coupled plasma mass spectrometry analysis. Covariates were assessed through validated questionnaires, in-person interviews, and medical record reviews. Conditional logistic regression was used to calculate multivariable-adjusted odds ratios (ORs). Results: Among women, the mean SSe concentration was lower in cases than in controls (98.5 μg/L vs. 103.8 μg/L; *p* = 0.016). In controls, SSe levels were directly associated with percentage of total energy intake from proteins and fish intake (*p* for linear trend < 0.001 and 0.049, respectively), whereas SSe concentrations were inversely associated with age, body mass index, and percentage of total energy intake from carbohydrates (*p* for linear trend < 0.001, 0.008 and 0.016 respectively). In the total group, we observed an inverse dose–response gradient between SSe levels and risk of CVD in the fully-adjusted model (highest vs. lowest quartile: OR = 0.47, 95% CI: 0.27–0.81; ptrend = 0.003). Conclusions: Among elderly individuals at high cardiovascular risk, high SSe concentrations within population reference values are associated with lower first CVD incidence.

## 1. Introduction

Selenium is an essential trace element which has been hypothesized as having a cardioprotective role due to the antioxidant properties of selenium-dependent glutathione peroxidases (GPxs) and other selenoproteins [1]. However, evidence from observational and experimental studies on the association between serum selenium (SSe) and cardiovascular disease (CVD) incidence and mortality has been reported as inconsistent.

Although selenium supplementation has been found to result in decreased levels of serum C-reactive protein (CRP) and increased GPx levels in randomized controlled trials [2], it has not been shown that selenium supplementation has a protective effect on CVD incidence [2,3,4,5] unless a low selenium status exists before selenium supplementation [6]. Moreover, because of the possible U-shaped relationship observed between selenium status and CVD risk [7,8], it is recommended to avoid excessive intake of selenium supplements, as this could increase selenium levels above an adequate range, potentially producing harmful effects [9,10].

Observational studies that have examined the relationship between plasma or SSe levels (the main indicator of actual selenium status [11]) and CVD incidence have found inverse associations only in populations with relatively low selenium status [12,13,14,15,16]. Recent studies have shown that even within reference ranges, SSe concentrations <100 μg/L can be considered suboptimal [17,18]. Nevertheless, observational studies in subjects without low selenium status did not show an association between selenium status and CVD incidence [7,19,20,21,22,23,24,25,26]. This lack of association may be due to the long follow-up periods considered in previous longitudinal studies, ranging from 5 to 25 years. Because selenium levels were measured at baseline in those longitudinal studies and selenium concentrations may change during the study periods [15], the time periods between SSe measurements and the onset of cardiovascular events may have been too long to accurately reflect the association between SSe levels and CVD. Moreover, as CVD pathogenesis may induce short-term changes in SSe levels, a long time frame between SSe determinations and cardiovascular events, as considered in previous studies, may be less appropriate to study these associations in participants at high cardiovascular risk. To the best of our knowledge, the association of SSe levels with near-term cardiovascular events has not previously been investigated.

Therefore, the aim of the present study within the “PREvención con DIeta MEDiterránea” (PREDIMED) trial [27,28] was to assess the short-term (<2 years) relationship between SSe levels and incident CVD risk in a population of Spanish adults aged 55–80 years who were at high risk of CVD.

## 2. Materials and Methods

### 2.1. Study Design

In the present study, data were analyzed using a paired-matched case-control design nested within the PREDIMED trial [28], a large randomized, multicentre, parallel-group, single-blind, and controlled CVD prevention trial that evaluated the effect of the Mediterranean diet (MedDiet) on the primary prevention of CVD. The trial included 7447 community-dwelling women and men ranging in age from 55 to 80 years at high cardiovascular risk and with no previously documented CVD. The study design and protocol have been described in detail elsewhere [27,29]. Participants were considered eligible (at high cardiovascular risk) in the presence of type 2 diabetes mellitus or at least three of the following risk factors: current smoking, hypertension (blood pressure > 140/90 mmHg or treatment with anti-hypertensive drugs), high plasma low-density lipoprotein (LDL)-cholesterol (>160 mg/dL or treatment with hypolipidemic drugs), low plasma high-density lipoprotein (HDL)-cholesterol (<50 mg/dL in women and <40 mg/dL in men), body mass index ≥ 25 kg/m^2^, or family history of premature CVD. Exclusion criteria included any severe chronic illness, history of CVD, drug or alcohol addiction, allergy or intolerance to olive oil or nuts, or low predicted likelihood of changing dietary habits according to the stages of change model, among others. From October 2003 to June 2009, participants were randomly assigned to three intervention groups: a MedDiet supplemented with extra-virgin olive oil (VOO), a MedDiet supplemented with mixed nuts, or a low-fat diet (control group) consisting of advice to reduce fat intake according to the American Heart Association guidelines. The PREDIMED trial was registered at http://www.controlled-trials.com with the number ISRCTN35739639 (accessed on 15 September 2022), and it was conducted according to the ethical standards guidelines of the Helsinki Declaration. All procedures were approved by the Institutional Review Boards of all the recruitment centres. Participants agreed and provided their written informed consent to authorize the use of biological samples for biochemical measurements.

### 2.2. Ascertainment of Cases and Selection of Controls

The primary endpoint was a composite of major cardiovascular events, defined as myocardial infarction, stroke, or death due to a cardiovascular cause. Four different information sources were used to collect these events: repeated contacts with the participants, contacts with the general practitioners providing clinical care to the participants, yearly review of participants’ medical records, and consultations of the National Death Registry through the Spanish National Statistics Institute to assess the vital status, as well as the cause of death in deceased participants. All medical records related to the endpoints were examined by the endpoint adjudication committee, whose members were blind to the intervention, metrics, and patients’ identities. Only those endpoints that were definitively confirmed by the adjudication committee and that occurred between October 2003 and December 2010 were included.

During a mean follow-up of 4.8 years, a total of 288 major cardiovascular events were identified. Sixty-two of them were excluded from the analysis because they had no available serum samples. For each case, we used incidence density sampling to select two controls matched by sex, intervention group, and age (±2 years). Thirty-five participants (19 cases and 16 controls) were excluded because their serum samples were inadequate for mass spectrometry analysis (not enough sample volume or poor sample quality). No differences were found between cases included and those not included in the analysis with regard to lifestyle and baseline characteristics. Finally, the present study involved 207 cases and 436 matched controls.

### 2.3. Blood Sample Collection and Measurement of Serum Selenium

At baseline and years 1, 3, 5, and 6 (or final visit), blood samples were collected in the morning between 8 and 10 am and after an overnight fast of at least 8 h. Blood samples were processed to obtain serum and stored at −80 °C at each recruiting centre. To reduce bias and inter-assay variability, samples from case-control pairs were randomly sorted and analysed in the same batch. Laboratory technicians were blinded to the interventions. For each case, we measured SSe by selecting the serum sample closest to (preceding) the cardiovascular event date in order to assess short-term risk. Median time between blood sample collection and subsequent CVD event occurrence was 0.94 years (interquartile range (IR): 0.38–1.96 years). Serum samples from controls were selected with the same follow-up period of the corresponding matching case (risk set sampling).

For the analysis of SSe, a calibration curve was prepared using a 1000 µg/mL Se standard solution (High-Purity Standards, Charleston, SC, USA) in a solution with 2% (*w/v*) 1-butanol (Merck, Darmstadt, Germany), 0.05% (*w/v*) EDTA (Aldrich, St. Louis, MO, USA), 0.05% (*w/v*) Triton X-100 (Merck, Darmstadt, Germany), and 1% (*w/v*) NH4OH (Merck, Darmstadt, Germany) in ultrapure water (Milli-Q, Merck, Darmstadt, Germany). Serum samples were diluted 1:10 in the above-mentioned solution. Metal analysis was performed on an Agilent 8900 triple-quadrupole inductively coupled plasma-mass spectrometer (Agilent Technologies, Santa Clara, CA, USA). The instrument was tuned and the performance parameters checked prior to analysis. To ensure the quality of the results, 40 µg/L germanium (ISC Science, Oviedo, Spain) was added to the samples as an internal standard. Furthermore, a suitable certified reference material (Seronorm (Sero, Billingstad, Norway) Trace Elements Serum L2 (reference 203105)) was reanalysed together with a blank and an intermediate calibration standard every twelve samples. National Institute of Standards and Technology NIST (USA) Trace Elements in Natural Water Standard Reference Material SRM 1640a was used as a certified reference material and analysed at the beginning and end of each sequence. Additionally, one of every twelve samples was reanalysed at the end of each session. Repeated re-measurement of a certificate standard serum sample yielded an intra-assay coefficient of variation of 4.35% and an inter-assay coefficient of variation of 2.67%. The limit of detection for selenium was 0.24 µg/L, and there were no concentrations bellow the limit of detection.

We used baseline fasting plasma samples to determine participants’ glucose, triglyceride, total cholesterol, LDL-cholesterol, and HDL-cholesterol. The Friedewald formula was used to calculate LDL-cholesterol levels whenever triglyceride levels were <300 mg/dL.

### 2.4. Covariate Assessment

During a face-to-face interview, qualified trained nutritionists administered a 47-item questionnaire about sociodemographic variables, lifestyle habits, disease prevalence, family histories of diseases, and medication use. Medications used habitually by participants were grouped into eight categories: angiotensin-converting enzyme inhibitors; diuretics; statins; insulin; aspirin-antiplatelet drugs; calcium channel blockers; angiotensin II receptor antagonists; and beta-blockers. The adherence to the MedDiet was assessed using a 14-item validated questionnaire [30]. A semi-quantitative 137-item food-frequency questionnaire that had been previously validated [31] was applied. For the present study, consumption of the following food groups was considered: legumes, grains, sweets and pastries, meat, dairy, fish, VOO, nuts, vegetables, fruits, and wine. Nutrients and energy intake were calculated according to the Spanish food composition tables [32]. The validated Spanish version of the Minnesota Leisure-Time Physical Activity Questionnaire [33,34] was used to assess physical activity. Trained and experienced personnel made anthropometric measurements, and blood pressure was measured according to the study protocol. At baseline and at each annual visit, blood pressure and anthropometric variables were measured, then the questionnaires were administered.

Hypertension, hypercholesterolemia, and diabetes were coded as binary variables (yes/no) based on the questionnaires and blood pressure measurements according to the following criteria: presence of hypertension if blood pressure > 140/90 mmHg or treatment with antihypertensive drugs prescribed by a physician; presence of hypercholesterolemia if participant has been diagnosed with hypercholesterolemia within the last year or treatment with anti-lipidemic drugs prescribed by a physician; presence of diabetes if participant has been diagnosed with type 2 diabetes mellitus. The above three binary variables were considered at the same time point as SSe measurement.

### 2.5. Statistical Analyses

Means and standard deviations (SDs) were used to describe quantitative variables and percentages were used to describe categorical variables. Group comparisons were carried out using *t*-test or chi-squared test as appropriate.

SSe levels (μg/L) were categorised in quartiles using the distribution among controls, then we applied the cut-off points to cases [35]. We used analysis of variance to assess adjusted levels of covariates across quartiles of SSe in controls. Associations of such covariates with quartiles of SSe were evaluated using polynomial contrasts (linear or quadratic trend).

We used conditional logistic regression models (conditional on the matching) to estimate the association between SSe levels and incident CVD. Three models with successive degrees of adjustment were estimated: (1) no adjustment, only with matching factors; (2) with adjustment for cardiovascular risk factors and potential confounders based on clinical relevance and previous causal knowledge: recruitment centre (indicator variables), current smoker (binary), hypertension (binary), hypercholesterolemia (binary), diabetes (binary), family history of premature coronary heart disease (binary), body mass index (kg/m^2^), and alcohol intake (g/day); and (3) with additional adjustment for adherence to MedDiet score (0–14 points), physical leisure activity (METs-min/day), total energy intake (kcal/day), intake of meat (g/day), fish (g/day), fruit (g/day), and VOO (g/day), percentage of total energy intake from carbohydrates (continuous), and use of calcium channel blockers (binary). The fully-adjusted model was built to include all these variables, showing a statistical association with SSe levels at *p* < 0.25 [36] as measured at sample collection time and without multicollinearity. Quartiles of SSe were included in the models as categorical variables, and we estimated the odds ratios (ORs) and 95% confidence intervals (Cis) for the three upper quartiles using the first quartile as the reference category. To analyse linear trends across the quartiles of SSe, we assigned the median value to each category and introduced it into the models as a continuous variable.

Potential interactions between quartiles of SSe and effect modifiers, such as sex, age, and intervention group (MedDiet supplemented with VOO, MedDiet supplemented with mixed nuts, or low-fat diet), were tested by adding a multiplicative interaction term (median value of the quartile of SSe x effect modifier) in the fully-adjusted model. The *p* values for the interactions were calculated using the likelihood ratio test based on the models with and without the interaction terms.

All statistical tests were two-sided with and α-level of 0.05. Statistical analyses were performed using Stata 15.1 (Stata Corp).

## 3. Results

### 3.1. Characteristics of Participants at Time of Blood Collection

The current study enrolled 643 participants (207 cases and 436 controls) with a mean age ± SD of 71.2 ± 6.7 years at sample collection time, of whom 398 (61.9%) were males. The mean level of SSe was 104.2 ± 16.4 μg/L. Mean SSe levels were significantly higher in men than in women (105.5 ± 16.6 μg/L vs. 102.1 ± 15.9 μg/L; *p* = 0.010). Table 1 presents the main characteristics of the study population at the time of sample collection. Among women, though not among men, the mean SSe concentration was lower in cases than in controls (98.5 μg/L vs. 103.8 μg/L; *p* = 0.016). Compared with control subjects, cases had a greater proportion of current smokers (20.3% vs. 11.9%; *p* = 0.005) and a higher prevalence of hypercholesterolemia (44.0% vs. 35.3%; *p* = 0.035), hypertension (61.8% vs. 50.9; *p* = 0.009), and diabetes (62.8% vs. 53.2%; *p* = 0.022) than controls. Concerning dietary intake, we found a non-significant lower adherence to MedDiet in cases than in controls (9.4 vs. 9.8; *p* = 0.058). The use of statins was significantly less frequent in cases than in controls (25.1% vs. 38.5%; *p* = 0.001), whereas a higher percentage of cases used aspirin-antiplatelet drugs compared to controls (33.8% vs. 22.0%; *p* = 0.001). At baseline, cases had higher average levels of glucose (137.7 vs. 124.2; *p* = 0.003) and triglycerides (147.6 vs. 130.4; *p* = 0.017) than controls.

### 3.2. Lifestyle and Dietary Factors Associated with SSe in Controls

The age-, sex- and centre-adjusted characteristics of controls according to quartiles of SSe are summarized in Table 2. Controls with higher selenium levels were more prone to be younger (*p* for linear trend < 0.001) and with a lower BMI (*p* for linear trend = 0.008). SSe levels were directly associated with percentage of total energy intake from proteins (*p* for linear trend < 0.001) and fish intake (*p* for linear trend = 0.049), and were inversely associated with percentage of total energy intake from carbohydrates (*p* for linear trend = 0.016).

### 3.3. SSe and Risk of CVD

Table 3 shows the associations of SSe levels with risk of CVD. In the unadjusted conditional logistic regression model, no significant associations between SSe levels and risk of CVD were found. Adjustment for cardiovascular risk factors and potential confounders revealed that participants in the third and fourth quartiles of SSe presented a significantly lower risk of developing CVD than those in the first quartile (OR = 0.55, 95% CI: 0.33–0.93 and OR = 0.54, 95% CI: 0.33–0.91, respectively; *p* for trend = 0.012). After full adjustment for all selected covariates, the associations between quartiles of SSe and CVD were enhanced (OR = 0.49, 95% CI: 0.28–0.83 and OR = 0.47, 95% CI: 0.27–0.81 for the third and fourth quartile, respectively, vs. the first quartile; *p* for trend = 0.003); *p*-values for interaction between SSe and potential effect modifiers were 0.391 for sex, 0.144 for age, 0.975 for MedDiet + VOO intervention arm, 0.387 for MedDiet + nuts intervention arm, and 0.222 for the low-fat diet group.

## 4. Discussion

In the present study, an inverse relationship was observed between selenium levels in serum and the incidence of CVD in a Mediterranean cohort with high risk of CVD. This is the first study, to the best of our knowledge, to show this relationship among asymptomatic participants without low selenium status.

It is known that selenium deficiency is associated with CVDs, such as Keshan disease, an endemic cardiomyopathy observed in low-selenium areas of China [37]. This, coupled with the capabilities of selenoproteins to reduce oxidative stress, prevent oxidative modification of lipids, inhibit platelet aggregation, and reduce inflammation [38], leads us to think that an inverse association between selenium status and CVD incidence may exist. Nevertheless, such an inverse association has been found only in epidemiological studies on populations with low selenium status, and evidence on selenium’s protective effects on incidence of CVD in addition to those observed on populations with suboptimal selenium status is inconclusive [4,10]. The population reference values (PRV) of SSe concentrations may be considered to be between 70–150 μg/L [39]. In our study, only eight participants (four cases and four controls) showed SSe levels below the PRV, while seven participants (three cases and four controls) presented SSe levels above the PRV. After excluding them from the analysis, our findings did not materially change (data not shown). Thus, our results support the idea that low selenium status increases the odds of CVD even in people with selenium status within PRV.

A main implication of our results is the cardiovascular benefit of maintaining an optimal level of selenium among people at high risk of CVD. Selenium status varies widely in different parts of the world, as diet is the primary source of selenium content in the human body and selenium food content is influenced by geographical location, seasonal changes, protein content, and food processing [40]. Therefore, it is complex to determine worldwide reference intervals for selenium status, and they should be locally established considering local diet. Nevertheless, peripheral selenium levels needed for optimal expression of selenoproteins with antioxidants capabilities such as GPxs and selenoprotein P [38] have been estimated in several studies. Plasma selenium concentrations ranging between 110 and 135 μg/L correspond to the plateau of platelet GPxs activity [41], and a mean plasma selenium concentration of 124 μg/L is associated with an optimal selenoprotein P level [42]. The above selenium concentrations are close to those observed in our study on participants in the highest quartile of SSe (mean = 125.3 μg/L, SD = 11.3 μg/L), whose cardiovascular risk was 53% lower. Thus, our results provide evidence on the importance of maintaining high selenium levels, even among people with a selenium status within reference levels. In fact, previous authors have proposed that SSe levels <100 μg/L should be considered as a certain selenium deficiency, or at least a suboptimal status [17,18]. Our results support the above, as we found a significantly lower risk of cardiovascular events only in participants in the third and fourth quartile, whose SSe levels were above the proposed suboptimal concentration. Therefore, among people at high cardiovascular risk, it would be advisable to increase selenium status in selenium deficiency situations as well as in those who, despite having selenium levels within PRV, do not reach the above optimal status. In addition to reducing CVD risk, maintaining SSe levels near 122 μg/L could likely reduce the risk of other diseases such as cancer and type-2 diabetes [38].

To safely increase selenium levels in populations with adequate selenium status, it is preferable to incorporate selenium-rich foods into the diet rather than supplements, as selenium can be toxic when its intake exceeds the organism’s capacity to eliminate it and the gap between deficient and toxic concentrations is narrow. Moreover, an increased risk of diabetes and hypercholesterolemia has been shown in randomized trials of selenium-containing supplements [43], and a U-shaped relationship has been observed between selenium concentrations and several diseases as well as mortality [44]. Based on our results (Table 2), a higher intake of fish and protein-rich foods and a lower intake of carbohydrates may be associated with an increase in SSe levels. Our observed associations between food intake and SSe levels agree with those described in previous studies, and an adequate intake of fish and meat has been recommended to improve selenium status and health in elderly people [45].

Our findings should be interpreted in proper context, as the suggestion that low SSe levels are associated with a higher risk of short-term CVD, even within reference range, has potentially significant clinical and public health implications. Our cohort consisted of elderly people at high cardiovascular risk with SSe levels within PRV. Preventive strategies should be a priority in these populations; therefore, the use of SSe concentrations as an independent risk indicator for incident cardiovascular events in older people free from CVD may be useful to reduce primary events in people at high risk. There may be several pathogenetic pathways of CVD that may affect SSe levels in a subclinical stage of the disease, when patients are asymptomatic. Thus, a decrease in SSe concentrations in elderly individuals should be considered as both an age-related fact and as a marker in the pathogenesis of CVD that should alert clinicians. Therefore, our findings underline the importance of monitoring selenium status for cardiovascular health in people at high cardiovascular risk. Further studies assessing the association between longitudinal changes in SSe levels (ideally using repeated measures) and CVD incidence could confirm the clinical utility of SSe concentrations as a marker of cardiovascular risk.

We acknowledge that several limitations and weaknesses should be highlighted in the current study. First, the participants were elderly subjects living in a Mediterranean region and at high cardiovascular risk; thus, it may not be possible to extrapolate the results to other populations. Second, although our analyses were extensively adjusted for potential confounders, due to the observational nature of our study we cannot exclude the possibility of residual confounding. Third, participants in our study underwent a nutritional intervention which might have affected both the incidence of CVD and SSe levels. We have minimized this effect by considering intervention group as a matching variable. Fourth, the use of supplements containing minerals/trace elements has not been assessed in our study. Finally, we have performed a single SSe measurement, which may be affected by within-individual variability, even though the PREDIMED trial was conducted with a well-designed protocol and quality control to minimize such variability [27]. In the absence of widely accepted and validated SSe specific cut-off points, we used quartiles as an acceptable and unbiased alternative for categorization of exposure. The single measurement of Se may yield random measurement errors, which tend to attenuate risk estimates. As a result, the inverse association of SSe with CVD is likely to be underestimated.

In addition, our study has important strengths. The nested case-control design presents considerable logistic and economic advantages because it uses existing cohort data and provides access to prospectively collected information. Data quality is high, as our study was built on a large trial with >4 years of follow up, a well-characterized population, an accurate and blind assessment of incident CVD cases, and controlled for a large number of potential confounding variables obtained from in-person visits. Blood samples were always drawn in the morning after a fasting period (≥8 h) and around the same hour. Moreover, parameter estimation and multivariate models were adjusted for centre to take into account possible differences in the handling of samples. The use of incidence density sampling minimized the possibility of control selection bias. We measured selenium concentration in serum, which is the most useful tissue for assessing actual selenium status [11,46]. Moreover, we used inductively coupled plasma mass spectrometry, which is the procedure of choice [11]. Finally, the short period between SSe measurement and event occurrence (<2 years) allowed for short-term assessment of CVD risk.

## 5. Conclusions

Within the population reference range, our results indicated that high SSe concentrations were associated with lower CVD incidence in a population at high cardiovascular risk irrespective of other classic cardiovascular risk factors. These findings highlight the importance of maintaining adequate selenium status and the utility of monitoring SSe levels as an independent marker of short-term risk in the overall assessment of primary CVD risk in these populations.

## Figures and Tables

**Table 1:** Characteristics of cases and matched controls at sample collection time. The PREDIMED trial.

Characteristic	Case Participants *n* = 207	Control Participants *n* = 436	*p* Value
Age (years)	70.9 (6.8)	71.3 (6.6)	MF
Sex (% women)	37.2	38.5	MF
*PREDIMED trial arm (%)*			
Mediterranean diet + Extra-VOO	32.4	31.0	MF
Mediterranean diet + nuts	27.1	27.5	MF
Serum selenium (μg/L)	102.9 (17.1)	104.9 (16.0)	0.143
Men (*n* = 401)	105.5 (17.7)	105.6 (16.0)	0.937
Women (*n* = 249)	98.5 (15.1)	103.8 (16.0)	**0.016**
*Smoking status (%)*			
Current	20.3	11.9	**0.005**
Former	37.2	34.2	0.453
Hypercholesterolemia (%)	44.0	35.3	**0.035**
Hypertension (%)	61.8	50.9	**0.009**
Type 2 diabetes (%)	62.8	53.2	**0.022**
Family history of CHD (%)	19.8	20.9	0.755
Body mass index (kg/m^2^)	29.5 (3.6)	29.2 (3.4)	0.305
Physical activity (METs-min/day)	239.9 (238.4)	275.6 (262.3)	0.097
Alcohol (g/day)	8.8 (15.1)	10.8 (14.6)	0.117
Glucose ^1^ (mg/dL)	137.7 (52.0)	124.2 (36.9)	**0.003**
Triglycerides ^1^ (mg/dL)	147.6 (82.6)	130.4 (60.7)	**0.017**
Total cholesterol ^1^ (mg/dL)	202.4 (33.4)	205.4 (38.9)	0.453
HDL cholesterol ^1^ (mg/dL)	49.0 (10.1)	50.7 (9.8)	0.107
MedDiet adherence (0 to 14)	9.4 (2.1)	9.8 (2.0)	0.058
*Dietary intake*			
Total energy intake (kcal/day)	2301.3 (646.2)	2283.3 (560.6)	0.717
Total Fat (%E)	40.1 (6.8)	40.0 (6.6)	0.951
Monounsaturated Fat (%E)	20.3 (4.5)	20.5 (4.4)	0.611
Polyunsaturated Fat (%E)	6.5 (2.1)	6.5 (2.0)	0.889
Saturated Fat (%E)	9.8 (2.3)	9.5 (2.1)	0.096
Carbohydrates (%E)	40.9 (7.3)	40.7 (6.9)	0.680
Proteins (%E)	16.5 (3.0)	16.2 (2.7)	0.191
Cholesterol (mg/day)	373.4 (142.7)	358.1 (129.4)	0.176
Fiber (g/day)	25.3 (9.3)	25.4 (7.7)	0.843
Legume (g/day)	23.6 (15.5)	22.5 (14.4)	0.363
Grains (g/day)	225.7 (104.3)	228.7 (111.4)	0.750
Sweets and pastries (g/day)	22.8 (36.4)	19.0 (24.3)	0.125
Meat (g/day)	132.3 (62.8)	124.8 (51.7)	0.111
Dairy (g/day)	381.2 (214.9)	357.8 (215.4)	0.196
Fish (g/day)	100.9 (50.9)	104.0 (46.4)	0.441
VOO (g/day)	44.0 (18.8)	45.1 (18.4)	0.469
Nuts (g/day)	13.7 (17.3)	14.7 (18.0)	0.495
Vegetables (g/day)	314.7 (133.5)	319.2 (140.7)	0.702
Fruits (g/day)	383.1 (207.7)	392.0 (181.1)	0.577
Wine (ml/day)	65.2 (113.5)	77.1 (113.7)	0.214
*Educational level (%)*			
Primary or less	77.8	79.0	0.941
Secondary	14.0	13.5	
Tertiary	8.2	7.6	
*Medication use (%)*			
ACE inhibitors	34.8	30.7	0.304
Diuretics	20.3	22.3	0.573
Statins	25.1	38.5	**0.001**
Insulin	7.7	5.5	0.275
Aspirin-antiplatelet drugs	33.8	22.0	**0.001**
Calcium channel blockers	19.3	15.4	0.208
Angiotensin II receptor antagonists	17.4	19.5	0.524
Beta-blockers	13.5	9.9	0.166

Data are provided as mean (standard deviation) or %. Statistically significant results are shown in bold (*p* < 0.05). MF: matching factor; PREDIMED: PREvención con Dieta MEDiterránea; VOO: virgin olive oil; CHD: coronary heart disease; METs: metabolic equivalents; MedDiet: Mediterranean diet; %E: percentage of total energy intake; ACE: angiotensin converting enzyme inhibitors. ^1^ Basal measurement.

**Table 2:** Adjusted ^1^ characteristics of 436 controls by quartiles of serum selenium at sample collection time.

Variables	Quartiles ^2^ of Serum Selenium				
	Q1	Q2	Q3	Q4	*p* for Linear Trend
No. of participants	109	109	109	109	
Median serum selenium level (μg/L)	86.60	99.23	109.58	121.77	NA
Men (*n* = 268)	87.20	99.34	109.18	121.30	NA
Women (*n* = 168)	86.23	99.03	109.89	124.16	NA
Age ^3^ (years)	73.68	70.67	70.81	70.19	**<0.001**
Sex ^4^ (% women)	38.00	41.61	44.31	30.22	0.321
*PREDIMED trial arm (%)*					
MedDiet + Extra-VOO	29.11	32.99	24.74	37.01	0.446
MedDiet + nuts	30.29	26.71	27.68	25.42	0.488
*Smoking status (%)*					
Current	11.14	17.57	12.74	6.25	0.156
Former	39.26	31.00	38.34	28.10	0.142
Hypercholesterolemia (%)	32.97	37.54	32.69	39.09	0.410
Hypertension (%)	53.42	54.91	45.99	49.35	0.331
Type 2 diabetes (%)	45.82	49.26	62.70	55.07	0.056
Family history of CHD (%)	22.41	19.98	21.01	20.08	0.735
Body mass index (kg/m^2^)	29.73	29.52	29.19	28.55	**0.008**
Physical activity (METs-min/day)	276.70	241.92	263.96	319.96	0.176
Alcohol (g/day)	11.40	10.58	9.74	11.42	0.891
Glucose ^5^ (mg/dL)	122.96	118.71	124.15	130.91	0.136
Triglycerides ^5^ (mg/dL)	131.31	127.81	128.54	134.02	0.786
Total cholesterol ^5^ (mg/dL)	207.75	199.72	208.55	204.72	0.989
HDL cholesterol ^5^ (mg/dL)	50.97	49.80	51.03	50.97	0.809
MedDiet adherence (0 to 14)	9.87	9.55	9.63	10.04	0.062 ^6^
*Dietary intake*					
Total energy intake (kcal/day)	2340.83	2270.25	2300.03	2221.97	0.172
Total Fat (%E)	40.00	39.33	40.46	40.38	0.406
Monounsaturated Fat (%E)	20.60	20.23	20.54	20.55	0.916
Polyunsaturated Fat (%E)	6.36	6.29	6.70	6.54	0.274
Saturated Fat (%E)	9.67	9.09	9.62	9.65	0.581
Carbohydrates (%E)	41.26	41.86	39.91	39.65	**0.016**
Proteins (%E)	15.50	15.66	16.83	16.65	**<0.001**
Cholesterol (mg/day)	361.49	333.16	376.08	361.64	0.438
Fiber (g/day)	25.89	24.95	25.95	24.78	0.488
Legume (g/day)	21.64	22.30	23.47	22.59	0.521
Grains (g/day)	240.87	235.48	219.98	218.34	0.080
Sweets and pastries (g/day)	20.48	19.59	19.10	17.00	0.298
Meat (g/day)	118.33	121.02	130.63	129.29	0.055
Dairy (g/day)	353.35	337.40	384.09	357.24	0.533
Fish (g/day)	98.89	96.55	114.56	106.07	**0.049**
VOO (g/day)	47.55	45.58	44.07	43.33	0.060
Nuts (g/day)	15.38	12.39	16.98	14.19	0.896
Vegetables (g/day)	317.87	315.34	340.19	303.40	0.761
Fruits (g/day)	419.62	386.99	388.65	374.93	0.110
Wine (mL/day)	78.11	75.41	72.95	81.98	0.845
*Educational level (%)*					
Primary or less	84.08	77.15	73.94	80.44	0.419
Tertiary	4.81	6.59	11.38	7.49	0.265
*Medication use (%)*					
ACE inhibitors	29.50	26.76	38.91	27.77	0.729
Diuretics	23.50	22.93	19.62	22.94	0.781
Statins	35.16	41.96	32.52	44.48	0.383
Insulin	8.93	0.93	7.37	4.78	0.549
Aspirin-antiplatelet drugs	18.69	26.68	22.58	20.12	0.992
Calcium channel blockers	13.27	15.12	12.12	20.96	0.202
Angiotensin II receptor antagonists	16.94	26.67	16.59	17.78	0.662
Beta-blockers	8.53	9.23	11.19	10.49	0.549

Data are provided as mean or %. Statistically significant results are shown in bold (*p* < 0.05). NA: not applicable; PREDIMED: PREvención con Dieta MEDiterránea; MedDiet: Mediterranean diet; VOO: virgin olive oil; CHD: coronary heart disease; METs: metabolic equivalents; %E: percentage of total energy intake; ACE: angiotensin converting enzyme inhibitors. ^1^ Adjusted for age, sex, and centre. ^2^ Quartiles of serum selenium based on distribution among controls. Cut-off values for quartiles of serum selenium were: 94.24, 104.03 and 115.03 μg/L. ^3^ Adjusted for sex and centre. ^4^ Adjusted for age and centre. ^5^ Basal measurement. ^6^ *p* for quadratic trend.

**Table 3:** Adjusted odds ratios for cardiovascular disease by quartiles of serum selenium. PREDIMED trial.

Variables	Quartiles ^1^ of Serum Selenium (μg/L)				
	Q1 (<94.3)	Q2 (94.3–104.0)	Q3 (104.1–115.0)	Q4 (>115.0)	*p* for Trend
Cases/matched controls	72/109	44/109	44/109	47/109	
Median serum selenium (μg/L)	87.14	99.66	109.74	121.51	
Matched OR ^2^ (95% CI)	1 (Ref.)	0.66 (0.41–1.05)	0.62 (0.39–1.00)	0.68 (0.43–1.07)	0.067
Matched OR ^3^ (95% CI)	1 (Ref.)	0.62 (0.37–1.04)	**0.55** **(0.33–0.93)**	**0.54** **(0.33–0.91)**	**0.012**
Matched OR ^4^ (95% CI)	1 (Ref.)	0.59 (0.34–1.00)	**0.49** **(0.28–0.83)**	**0.47** **(0.27–0.81)**	**0.003**

Statistically significant results are shown in bold (*p* < 0.05). ^1^ Cut-off points based on distribution among controls. ^2^ Models are from conditional logistic regression analyses with matching factors sex, age, and intervention group. ^3^ Adjusted for centre (indicator variables), smoking (binary), hypertension, hypercholesterolemia, diabetes, family history of premature coronary heart disease, body mass index (continuous), and alcohol intake (g/day). ^4^ Additionally adjusted for adherence to the Mediterranean diet (0–14 points), physical activity (METs-min/day), total energy intake (kcal/day), meat intake (g/day), fish intake (g/day), fruit intake (g/day), virgin olive oil intake(g/day), percentage of total energy intake from carbohydrates (continuous), percentage of total energy intake from proteins (continuous), and calcium channel blocker use (binary).

## Data Availability

There are restrictions on the availability of data for the PREDIMED trial due to the signed consent agreements around data sharing, which only allow access to external researchers for studies following project purposes. Requestors wishing to access the PREDIMED-Plus trial data used in this study can make a request to the PREDIMED trial Steering Committee chair: restruch@clinic.cat. The request will then be passed to members of the PREDIMED Steering Committee for deliberation.
